# Treatment with CBA-NP a novel chimeric natriuretic peptide attenuates cardiorenal fibrosis and improves diastolic dysfunction in diabetic rat model

**DOI:** 10.1186/1471-2210-11-S1-P2

**Published:** 2011-08-01

**Authors:** Syed Ameenuddin, Elise A Oehler, John C Burnett, Horng H Chen

**Affiliations:** 1 Department of Internal Medicine, Division of Cardiovascular Diseases, Mayo Clinic, Rochester MN 55902, USA

## Introduction

Diabetes is a major risk factor for left ventricular dysfunction with cardiac and renal fibrosis. C-type natriuretic peptide (CNP) is a 22 amino-acid peptide produced mainly in the cardiac endothelium with potent cardiac unloading, anti-fibrotic and antihypertensive effects, but minimal renal actions. Using this knowledge we designed a natriuretic peptide CBA-NP by fusing a 6 AA sequence (KVLRRH) from BNP to the C-terminus and a 5 AA sequence (RMDRI) from ANP to the N-terminus of CNP to enhance beneficial renal effects while maintaining CNP’s inherent cardioprotective properties.

## Hypothesis

Chronic treatment with CBA-NP will have direct anti-fibrotic and humoral effects in a rat model of diabetic cardiomyopathy.

## Methods

Using three groups of six male Wistar rats, (normal control, diabetic control, and diabetic treated with CBA-NP) one dose of streptozotocin was administered to induce diabetes. One month after induction of diabetes ALZET pumps with 0.1μg/kg/min of CBA-NP or saline were serially implanted subcutaneously every 14 days over the course of 2 months. Cardiac function was assessed by echocardiography. Neurohormones by RIA. Fibrosis by picrosirius red staining. Ultrastructural features by electron microscopy.

## Results

CBA-NP treatment attenuated LV hypertrophy (0.24±.01 mg/g body weight) compared to diabetic control (0.26±.01) and was comparable to the normal control (0.24±.01). LV interstitial and perivascular fibrosis percentage was significantly reduced in the CBA-NP treated group (3.27 to 1.80 and 3.87 to 1.77) as compared to the diabetic control. Ejection fraction (84.0± 1.2% vs. 78.0±1.7 %) and fractional shortening (48±1.2% vs. 41±1%) were significantly improved after CBA-NP treatment compared to diabetic control. Kidney cortical and medullary percent fibrosis was significantly reduced (4.58±0.80 to 2.16±0.14 and 4.43±0.6 to 1.23±0.3) after CBA-NP treatment as compared to the untreated group. GFR significantly improved (1.74±0.18 to 2.42±0.18) with reduction in glomerular basement membrane thickness. There was a significant decrease in plasma renin, aldosterone and BNP, while plasma cGMP increased in the treated group compared to the untreated group.

## Conclusion

CBA-NP treatment attenuated LV hypertrophy, reduced cardiac and renal fibrosis, and improved cardiac and renal function with suppression of renin and aldosterone, suggesting a potential therapeutic benefit in diabetic cardiomyopathy.

